# Supplementary material for: To participate or not to participate? A qualitative investigation of students’ complex motivations for verbal classroom participation
Source: PLoS One. 2024 Feb 6;19(2):e0297771. doi: 10.1371/journal.pone.0297771 (PMC10846701; doi:10.1371/journal.pone.0297771)
Supplement: S1 Appendix — (DOCX) [file pone.0297771.s001.docx]

INTERVIEW QUESTIONS

BACKGROUND

- (Age; Major; Class)
- How long have you attended the university?
- What is your major area of study? Why did you choose that major?
  - Was your choice influenced by your academic strengths, interests, or both?
- What do you intend to do after college?
  - Have you ever changed your major/career plans? Why?
  - How much do you think about future lifestyle choice or other life goals when considering academic plans?
- What makes you feel like you belong in your major and/or your classes? (synonyms: at home? Acceptance? In the right place? Where you should be?)
- What makes you feel like you don’t belong?
- What are the first reactions you usually get when you tell people your major and/or future career plans? How does that affect you?

PARTICIPATION

- How much do you typically participate verbally in your classes?
- What are the reasons you do participate in class? What are reasons you do not participate?
- Have there been classes that you are more willing to participate in than others? How does your participation vary from class? What about those classes made you participate more?
  - Anything about the instructor specifically? Anything about your peers? *(Note: Except in one instance where a woman participant was asked about professors without her bringing up professors organically, these follow-ups were only used to ask students to elaborate on instructors and peers after the interviewee had already mentioned them.)*
  - How do your bioinformatics courses compare to your other courses?
- Compare your experience in high school with your experience here at the university. Do you participate more or less? Why?
- Compare your experience participating in class with how vocal you tend to be in social situations. Do you participate more or less? Why?
- What other ways do you participate in class?

PERFORMANCE

- How would you rate your academic performance in comparison with your peers? Why do you think that?
  - “Natural” ability vs how hard you have to work
  - Talk about STEM courses versus others (What subjects do you consider to be your academic strengths?)
- How do think your peers view you? Do you think they see you as a high performer or a low performer?
- What factors do you believe influence your performance in a class? Why do some students perform better than others?
  - People around you? (friends, family, faculty)
  - ability, hard work, etc.
- What are the major roadblocks or obstacles students face in trying to succeed academically?
  - Do you think any of those roadblocks are different for different students? (females versus male students)
  - Differing expectations?
- What do you do if you don’t understand a concept in your classes?
- What classes have you been really successful in? Why do you think you were able to be so successful?
  - Was everyone successful in that class? Or were you more or less successful than your peers?
  - Natural vs had to work really hard?
  - What role did that specific instructor have on your success?
- Have you ever done poorly in a class (college or high school)? Why do you think that happened?
  - Ability vs work put in
  - Lack of resources available
  - If they just talk about a hard topic…
    - Do you think you struggled any more or less than your peers in that class? Why?
    - Did that experience change anything for you? How you studied in future classes? Your major?
  - What role did that specific instructor have on your experience?
- How would you define academic success overall? How do you measure your success?
  - Grades (A)
  - Understanding what you’re learning
  - Enjoying it
- Would you describe yourself as a successful student? How would you rate your success in comparison with your peers? Why do you think that?
- Has your definition of success changed since attending BYU? (high school vs college)

PARTICIPATION AND PERFORMANCE

We’ve already talked a lot about participation and performance in classes. I’m also interested in how they influence each other.

- If you think about classes where you did or did not participate a lot, and then classes you did well in and classes you struggled in, were they any relationships between those?
- In your opinion does participating in lectures improve a student’s success in a class?
- When you notice a student participates a lot in class, does that affect your opinion of their aptitude/experience/knowledge in the course subject?
