# Supplementary material for: To participate or not to participate? A qualitative investigation of students’ complex motivations for verbal classroom participation
Source: PLoS One. 2024 Feb 6;19(2):e0297771. doi: 10.1371/journal.pone.0297771 (PMC10846701; doi:10.1371/journal.pone.0297771)
Supplement: Attachment — Submitted filename: Response to Reviewers.docx [file pone.0297771.s002.docx]

**Response to Reviewers**

We recognize that you obtained ethical approval from your local ethics committee after you began the study: your ethics approval document is dated February 21, 2020, while participants were recruited beginning from June 2019 and April 2020. For human subjects research, PLOS ONE requires that authors obtain ethics committee approval before beginning the study, and we do not accept retrospective ethical approval.

Apologies for uploading the wrong IRB approval file with the original submission. We did receive IRB approval before participant recruitment began, and the original approval letter was dated April 19, 2019. That approval letter is now uploaded with the submission. We later modified the IRB protocol to allow Zoom interviews, and the modification approval letter was the file I accidentally included in the original submission.
