# Supplementary material for: To participate or not to participate? A qualitative investigation of students’ complex motivations for verbal classroom participation
Source: PLoS One. 2024 Feb 6;19(2):e0297771. doi: 10.1371/journal.pone.0297771 (PMC10846701; doi:10.1371/journal.pone.0297771)
Supplement: Attachment — Submitted filename: REVIEWERS REPORT.docx [file pone.0297771.s003.docx]

| **Reviewer:** Dr. P.A. Gerrand | |
| --- | --- |
| **Manuscript Number**: PONE-D-23-19417 | |
| **Article Type**: Research Article | |
| **Full Title:** To participate or not to participate? A qualitative investigation of students’ complex motivations for classroom participation | |
| **OVERALL COMMENTS** | This article is interesting and holds the potential to provide important information for instructors from different disciplines that want to enhance verbal participation by students in class.  Below are my suggestions regarding how the article can be improved. |
| **Title** | The title is simple and direct. However, consider inserting the word ‘verbal’ before the words ‘classroom participation because your article focuses specifically on verbal participation in the classroom. |
| **Line Number** |  |
| Ls 12 - 31 | **Abstract**  The abstract is written and structured well. It provides and introduction to the background of the research, methods used, results of the study, and conclusions reached. The researchers also inform the reader that limitations of the study are covered in the report and how the results could be framed within the Expectancy Value Theory of Motivation. |
| L 113 | Before using the abbreviation ‘STEM’ write what it stands for in full. This is because instructors from other disciplines can decide to read your article. |
| 119 | Clarify this comment (‘JS and one man who only participated in group discussions’). You do not mention that focus group discussions were conducted to gather results presented in this article. |
| 120 | Also clarify this comment: “10 women and 9 men responded, agreed to participate in the study, and showed up for the interview. However, in L 184 you point out that most of the interviews were conducted online using Zoom, but eight women were interviewed in person” |
| 143 | You mention that “Participants were not given the chance to comment on the transcriptions after they were complete.” Any reason why this did not take place because this procedure is frequently used to enhance the trustworthiness of qualitative research.  It was a good idea to have all three researchers analyse data gathered to improve trustworthiness of the study. |
| 206 - 654 | All three themes emerging do capture essence of data analysis. However, although the content of Themes 1 and 2 include many verbatim quotes to substantiate research findings, this does not take place when discussing Theme 3. (In fact, no verbatim quotes appear). The discussion seems to focus on important findings apparent in Themes 1 and 2 and how these are related to Theme 3. |
| L 217 | Figure 1 is visually appealing and summarises the main research findings.  However, it is presented in colour (i.e., different colours represent different research findings.) This is concerning because PLOS ONE prints figures and tables in greyscale. For this reason, you will have to explain what the figure represents a little more clearly. |
| L 663 | Be careful to correctly interpret out researchers’ findings. For example, you cite Mustapha, Abd Rahman and make the following comment:  “Depending on what the students meant by poor teaching skills  in that study, this could be validated by our finding that students valued instructors who could competently answer their questions, increasing their confidence that it would be worth it to ask their  questions. However, the author explains that students’ verbal participant was related to instructors’ personality traits and teaching style. S |
